# PRIMING OF MICROGLIA ACTIVITY INCREASES SUSCEPTIBILITY TO DEPRESSION-LIKE BEHAVIORS

**DOI:** 10.1093/geroni/igz038.359

**Published:** 2019-11-08

**Authors:** Tal Frolinger, Umar Iqbal, Giulio M Pasinetti

**Affiliations:** Icahn School of Medicine at Mount Sinai, New York, New York, United States

## Abstract

This study investigates the role of microglia activity in stress-induced depression and anxiety and the mechanisms associated with the role of certain microbiome derived anti-inflammatory polyphenols in attenuating stress-induced microglia immune priming and symptoms of depression. We implemented a chronic unpredictable stress (CUS) paradigm to exhibit priming of microglia innate immunity in the context of the onset of depression and anxiety phenotypes. Mechanistic studies related to prophylactic treatment using dietary microbiome derived polyphenols were also investigated in this model. Depression and anxiety phenotypes, gene expression in microglia and protein expression in the cortex of mice were measured following a primary exposure to short-term unpredictable stress (US) followed by CUS. We examined the long-term, persistent CUS induced changes at 4-weeks of post-stress rest following a secondary US exposure. We found depression phenotypes resulted from US only following exposure to CUS. This was accompanied by an increase and persistent upregulation of toll-like receptor 4 (TLR4), RAGE, and HMGB1 gene expression in isolated cortical microglia. Priming by CUS also amplified gene expression of IL-1β in microglia and protein IL-1β in the cerebral cortex following US re-exposure. Increased activity of NF-kB was also noted in the period following CUS. Furthermore, polyphenol treatment prevented stress-induced phenotypes, upregulation of HMGB1, IL-1B, and TLR4 gene expression, as well as upregulation of IL-1β and NF-kB. The study suggests that latent activity of the TLR4-NFkB-IL1β pathway contributes to immune priming and increases susceptibility to depression-like behaviors. Anti-depressant effects of polyphenols may result from their ability to attenuate microglia priming.

